# Impact of Linker Defects on the Dielectric Properties of the Metal-Organic Framework HKUST-1: Insights from Molecular Dynamics Simulations

**DOI:** 10.1021/acsami.6c03219

**Published:** 2026-06-19

**Authors:** Yidian Wang, Piero Macchi

**Affiliations:** Department of Chemistry, Materials and Chemical Engineering, 18981 Politecnico di Milano, Via Bassini 6, Milano 20133, Italy

**Keywords:** metal−organic framework, dielectric constant, missing linker defects, molecular dynamics simulation, polarizable force field

## Abstract

Ultra-low dielectric materials are essential for the modern microelectronics industry, and metal–organic frameworks (MOFs) are promising candidates for the next generation of such materials. In this work, we use molecular dynamics simulations and the generic UFF4MOF force field to predict how missing linker defects influence the dielectric properties of a very well-known MOF like HKUST-1. The thermalized Drude oscillators are introduced to develop the DO-UFF4MOF force field for the polarization response of the MOF framework. This polarizable force field is first benchmarked against the dielectric constant of pristine HKUST-1 and then applied to simulate derivatives containing missing linker defects. Our simulations suggest that a defect engineering approach could effectively reduce the dielectric susceptibility by up to ca. 30%, potentially achieving the lowest dielectric constant for this framework type (*κ* = 1.5). We observe a linear correlation between the dielectric constant and the concentration of linker defects, driven by a strong reduction in material polarizability that significantly outweighs the decrease in mass density. Furthermore, even low concentrations of missing linker defects substantially accelerate the dielectric relaxation.

## Graphical abstract

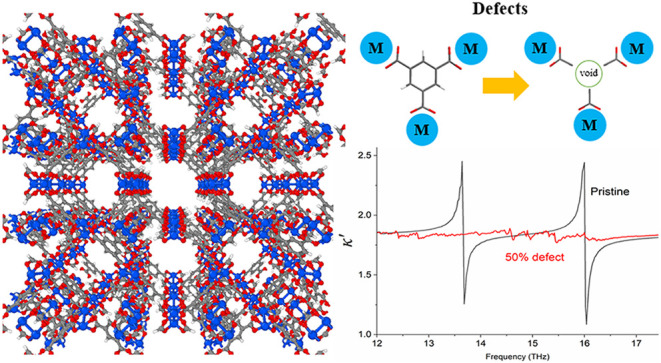

## 1 Introduction

The structure–property relationship involves the control of macroscopic behaviors through microscopic perturbations of the structure, making it a pivotal topic in materials science.^1–3^ In the microelectronics industry, the miniaturization of integrated circuits is approaching the nanometer (nm) scale at a rapid pace. However, persistent engineering challenges indicate that finding ultra-low dielectric constant (κ) materials to replace the traditional, but inefficient SiO_2_-based ones (κ ∼ 3.9) is vital. Metal-organic frameworks (MOFs)—constructed via the self-assembly of metal ions (or clusters) with organic ligands—are promising candidates for next-generation interlayer dielectrics because of their tunable void accessibility, their chemical, thermal, and mechanical stability, and their light composition, which guarantees a low polarizability of the material.^4–6^ Appropriately designed MOFs can be highly porous, inert, and insulating.

The dielectric properties of MOFs have attracted ever-increasing attention following the predictions made for a class of Zn_4_O­(CO_2_)_6_-based MOFs.^7^ Subsequently, several carboxylate-based frameworks were studied more accurately using density functional theory (DFT)^8^ to analyze the effects of organic linker functionalization and metal replacement. In 2019, our group experimentally and theoretically investigated the dielectric properties of highly porous Cu_3_(BTC)_2_ and Zn_3_(BTC)_2_ (where BTC = 1,3,5-benzenetricarboxylate), focusing on the nature and strength of water coordination in the apical positions of paddlewheel Cu dimers in the hydrated phases of those MOFs.^9^ Systematic frequency-resolved studies explored the polarization mechanisms in a series of MOFs and zeolitic imidazolate frameworks (ZIFs),^10–13^ while the dielectric response of the host–guest system was investigated in HKUST-1,^14^ Mn_3_(HCOO)_6_, lanthanide-based MOFs, and MIL-53­(Al).^15–18^ More recently, Jahromi et al. used molecular dynamics (MD) to show how the dielectric susceptibility of such systems depends on the nature of adsorbed molecules.^19^ Considerable effort has also been devoted to protecting MOFs from atmospheric water adsorption, for example, by functionalizing UiO-66 MOFs with external polydimethylsiloxane and internal linker modification^20^ or by preparing amine-protected HKUST-1.^21^ On a more practical level, Krishtab et al. reported a vapor-phase deposition strategy for integrating zeolitic imidazolate frameworks (ZIFs) as ultra-low-κ dielectrics in advanced on-chip interconnects.^22^

Less attention has been paid to the role of structural defects in the dielectric properties. Defects naturally occur in MOFs even with careful preparation because uncontrollable events during chemical reactions can create vacancies of metal nodes or organic linkers.^23,24^ On the other hand, defects can also be intentionally introduced to modify adsorption, catalytic activity, or conductivity.^25^ The most common methods of defect engineering are: (a) replacing ligands with lower hapticity acid modulators to create missing linker or cluster defects;^26^ (b) post-synthetic modifications via etching,^27,28^ and thermal or mechanical modulation;^29,30^ and (c) modulating nucleation kinetics during synthesis.^31^ *Ab initio* simulations typically assume perfectly crystalline MOF structures to reduce computational load, but defects disrupt regular periodicity and significantly affect MOF properties. Atomistic molecular dynamics simulation offers a reliable alternative because simulations with 10^4^ to 10^6^ independent atoms are feasible, making MD the elective method for defect engineering predictions. Islamov et al. calculated the thermal conductivity of HKUST-1 with elevated missing linker defect concentration at room temperature,^32^ while the effects of both randomly created missing linker and missing cluster defects were studied in UiO-66.^33^ The mechanical properties of missing linker-engineered HKUST-1 were studied by Wang *et al.* with ReaxFF (reactive force field) MD simulations.^34^ Similarly, UiO-66 with linker defects was simulated with UFF to investigate the relative stability.^35^

In this article, we focus on linker vacancies, a type of defect, and their effect on the dielectric properties of a widely studied MOF, [Cu_3_(BTC)_2_], also named HKUST-1. Using classical MD simulations combined with the finite element method, thermalized Drude oscillators, and the Thole screening scheme, we expect to reveal the structure–dielectric property relationship of defective MOFs from a microscopic perspective. For this goal, it is first necessary to optimize the damping factor of the polarizable force field (not tuned for MOFs) in order to accurately reproduce the static dielectric constant for HKUST-1. Afterward, we will be able to investigate how missing linker defects influence the dielectric constant. We expect to obtain both a correlation with structural attributes and an estimation of the lowest dielectric constant potentially achievable via defect engineering. Moreover, the frequency-dependent dielectric response will allow us to exploit the internal microscopic variations triggered by macroscopic alterations.

## 2 Methods

For the simulations, we used classical molecular dynamics due to the breakdown of the regular periodic arrangement, with the purpose of optimizing the structure of pristine and defective HKUST-1, accurately calculating the dielectric constant *κ* values using the finite element method by applying an external electric field, and investigating the impact on the frequency dependent dielectric spectrum. For this reason, we needed to optimize the Coulombic screening strength in the polarizable force field (FF), generate defective structure models for each selected composition, and refine the methods for the evaluation of κ­(ω).

### 2.1 Force Field

In MD simulation, the fixed-charge classical FF usually underestimates the dielectric constant of materials without permanent dipole moments.^36^ The Drude oscillator (DO) model offers a solution to this limitation:^37,38^ a Drude particle (DP) carrying part of the atomic charge is bound to the core atom (Drude core, DC) via a harmonic spring to mimic the relative motion of the nucleus and electrons. The motion of the DP is calculated by minimizing the energy of the induced dipoles at each timestep through an iterative, self-consistent procedure with an extended Lagrangian method. In the literature, attempts have been reported to integrate the DO model into fixed-charge classical FF. All of them are computationally stable and provide better performance compared to the original model.^39–41^ For the simulations of HKUST-1 systems, we integrated the DO model into the well-reckoned universal force field for MOF (UFF4MOF) and adopted the external field method. The functional takes the form:

1 $$U_{DO-UFF4MOF}\left(r_{ij}\right)=U_{UFF4MOF}\left(r_{ij}\right)+U_{\mathrm{self}}\left(r_{ij}\right)$$

where *U*_*UFF*4*MOF*_(*r*_*ij*_) is the UFF4MOF interatomic potential (eq S1); $U_{\mathrm{self}}\left(r_{ij}\right)=K_{D}{(r_{ij}^{D}-r_{0}^{D})}^{2}$ represents the bond between DC and DP with a harmonic oscillator, where $r_{ij}^{D}$ is the displacement of DP from DC at each step, $r_{0}^{D}$ is the equilibrium distance, and $K_{D}=\frac{1}{2}\frac{q_{D}^{2}}{\alpha }$ is the spring constant defined by the relation with atomic polarizability (*α*) and the DP charge (*q*_*D*_). It is important to note that UFF4MOF does not include atomic point charges. Thus, the atomic partial charges for the different interaction sites were fitted using the extended charge equilibration (EQeq) method, specifically designed to improve the description of the charge of the metallic nodes and the computational efficiency for supercell and non-symmetric models.^42^

### 2.2 Structural Model

The target of our investigations is the widely used MOF HKUST-1, which consists of dimeric paddlewheel nodes, with each copper dimer connected to four BTC linkers (Figure 1a) and each BTC connecting 3 nodes. Its crystal structure belongs to the *Fm3m* space group, featuring large voids and an overall empty space of ca. 70%. Exposure of pure HKUST-1 to a humid atmosphere reveals its highly hygroscopic nature: water molecules penetrate the pores and bind to the apical free position of the paddlewheels or occupy the empty channels. In our simulations, however, we assumed working with water-free species, thus in an inert atmosphere.

**Figure 1:**
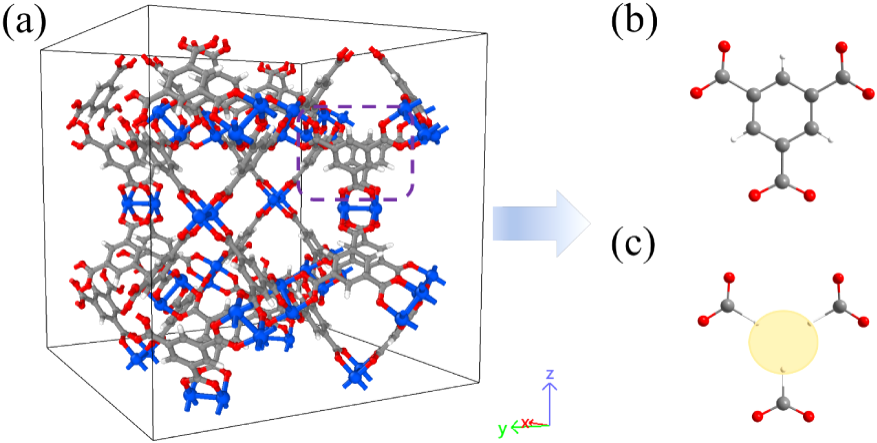
(a) Perspective view of one unit cell of pristine HKUST-1. The BTC linker, outlined with a purple square, is selected for defect introduction; (b) the BTC organic linker; (c) the local missing linker defect structure formed by replacing one BTC with three FA. The pale orange region in (c) addresses the free space created by removing 6 carbon atoms and creates ca. 120 Å^3^ of void. The color scheme for the atoms is as follows: copper (blue), carbon (brown), hydrogen (white), and oxygen (red).

As confirmed by experiments,^43^ missing linker defects can be induced by using formic acid (HFA) as a modulator. In our defect models, each defect site was created by replacing one BTC linker with three formate anions (FA), thus preserving charge neutrality in all of the systems under study (Figure 1). Each FA binds to only one Cu dimer, thus breaking the internode linkage. We simulated defect concentrations from 5% up to 50%, corresponding to the percentage of the removed BTC linkers. The metal nodes were, instead, kept identical to the pristine ideal structure (Figure 1b and 1c). To sample the phase space, three independent configurations with a random distribution of defect sites were generated for each defect degree, and the dielectric constant was averaged across those configurations. The linker removal and replacement were generated with PoreMatMod.jl using a graph-based find-and-replace principle.^44^

To evaluate the possible finite-size effects, we performed simulations using a 1 × 1 × 1 unit cell, as well as 2 × 2 × 2 and 3 × 3 × 3 supercells of pristine HKUST-1 before calculating the defective models.

### 2.3 Dielectric Constant

The dielectric constant of a material addresses its ability to be polarized in response to electrical stimuli. We can break down the polarization mechanism into different contributions: (i) electronic polarization; (ii) reorientation of permanent dipole moments; (iii) ionic (charge-transfer) polarization; and (iv) interfacial polarization involving the reorganization of space charges at interfaces.^45–48^ The first three terms are relevant to the dielectric constant of MOFs. Electronic polarization becomes increasingly dominant at very high frequencies, but it is not accounted for in classical MD with fixed-charge FF, whereas the other terms become progressively less significant with increasing frequency.^36^ For the static polarizability, electronic polarization is also the most important term.

The calculation of *κ* is based on linear response theory,^49,50^ assuming that the induced dipole depends linearly on the electric field E⃗^ext^. The time-dependent dielectric constant *κ_i_* (*t*) is then calculated as:

2 $$\kappa_{i}\left(t\right)=1+\chi_{i}\left(t\right)=1+\frac{4\pi }{V}\frac{\Delta \mu_{i}\left(t\right)}{E_{i}^{\mathrm{ext}}}$$

where *χ* is the dielectric susceptibility, and $\Delta \mu_{i}\left(t\right)=\mu_{i}^{E}\left(t\right)-\mu_{i}\left(t\right)$ , in which $\mu_{i}^{E}\left(t\right)$ and *μ_i_* (*t*) are the dipole moments with and without an external electric field $E_{i}^{\mathrm{ext}}$ in the direction *i*; *V* is the volume of the simulation box.

### 2.4 Simulation Protocol

We first optimized the ideally pure HKUST-1 crystal structure using periodic density functional theory (p-DFT) calculations. The hybrid DFT functional B3LYP and the pob-TZVP^51^ basis set were used, and the calculations were performed with CRYSTAL17.^52^

We then estimated the effect of linker replacement on HKUST-1 fragments [Cu_2_(H_2_BTC)_4_], [Cu_2_(H_2_BTC)_3_(FA)], [Cu_2_ *o*-(H_2_BTC)_2_(FA)_2_], [Cu_2_ *p*-(H_2_BTC)_2_(FA)_2_], [Cu_2_(H_2_BTC)­(FA)_3_], and [Cu_2_(FA)_4_] for which we calculated the polarizability α at B3LYP/6–311++g­(2d,2p)^53,54^ level using Gaussian 16.^55^ We partitioned α using the quantum theory of atoms in molecules,^56^ calculated with AIMAll,^57^ and used PolaBer for the calculation of the atomic tensors.^58^

The open-source package Large-scale Atomic/Molecular Massively Parallel Simulator (LAMMPS) was used for all MD simulations.^59^ The EQeq charges were assigned to atoms in the initial configuration (Scheme 1) for both pristine and defective models and kept fixed during simulations. Periodic boundary conditions with a cutoff for Lennard–Jones interactions of 12.5 Å were applied, and the electrostatic interactions were calculated through Ewald summations. Analytical tail corrections and the Lorentz–Berthelot mixing rules were applied for non-bonded interactions. The equations of motion were integrated using a Velocity-Verlet algorithm with a time step of 0.25 fs. The parameters of the DO model were adopted from the method developed by Lamoureux and Roux.^37^ The massless DP was assigned *m*_D_ = 0.4 g/mol, and the DC-DP pair was harmonically attached with a force constant *K*_D_ of 4184.0 kJ/mol/Å^2^ for all atoms, except for hydrogen atoms, which were treated as non-polarizable.

**Scheme 1:**
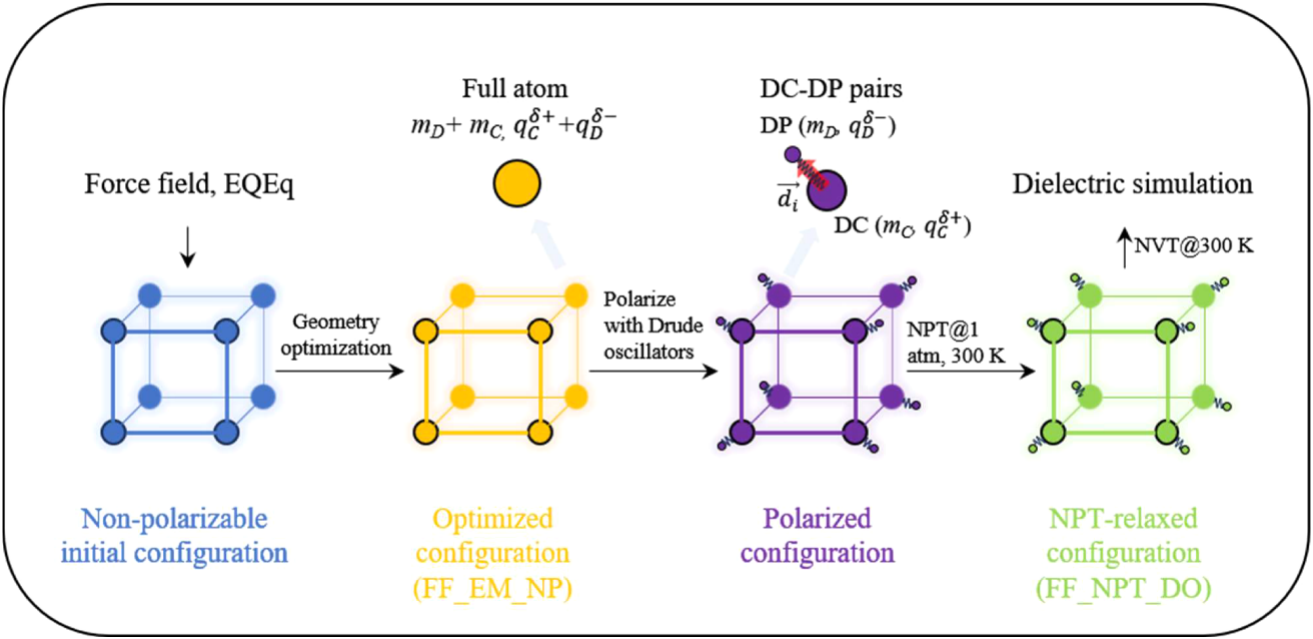
The Workflow of MD Simulation for the Calculation of Dielectric Constant of MOF^a^

a The cubic frameworks represent MOF structures, while different colors denote the corresponding configurations.

To fully relax the geometry with the DO-integrated UFF4MOF model, we adopted the following procedure (Scheme 1):

(1) A conjugate gradient algorithm was used to iteratively and simultaneously relax both the cell parameters and atomic positions, allowing for anisotropic relaxation at ambient pressure to find a stationary point on the energy surface of the charged non-polarizable (NP) UFF4MOF model. Using termination criteria for energy of 10^–10^ kcal mol^–1^ and for force of 10^–10^ kcal mol^–1^ Å^–1^, the obtained configuration was labeled as FF_EM_NP.
(2) The polarization treatment with Drude oscillators was applied to the FF_EM_NP model.
(3) A further relaxation step with NPT-MD was performed on the polarizable model to obtain an equilibrium configuration, hereby noted as FF_NPT_DO, and eventually used for the calculations of the dielectric properties.

Two independent Langevin thermostats were applied to thermalize the reduced degrees of freedom of DPs to 1 K, while DCs were kept at 300 K during all the simulations. The equilibration NPT run at a constant pressure of 1 atm for step (3) was conducted for 500 ps. After the system density reached stability, the dipole moments with and without a finite electric field were computed every 400 steps in parallel under the NVT ensemble for 100 ps. The strength of the applied electric field range was 0.025711 V/Å (0.005 au).

## 3 Results and Discussion

### 3.1 Benchmarking DO-UFF4MOF

As one would expect, the NP model predicts an ultra-weak dielectric response under a static electric field (Figure S1). As previously reported, the structure of HKUST-1 can be optimized with the original (non-polarizable) DDEC-charged UFF4MOF, with an error on the unit cell volume smaller than 10% error.^60^ Since the volume is inversely correlated with the dielectric constant, we need to validate the feasibility of incorporating the classical DO method and EQeq charge scheme in the UFF4MOF force field (Table S1), thereby achieving a good prediction of both the structure and the dielectric constant. The pristine HKUST-1 optimized geometry calculated with the NP model (FF_EM_NP in Scheme 1) has a volume ca. 6% larger than the p-DFT prediction (Table S2) and ca. 7.6% larger than that experimentally measured. The structure optimized with the model including the Drude oscillators does not differ from the NP model (Table S3). Overall, a consistent deformation trend is observed for the FF_NPT_DO model relative to FF_EM_NP when different supercells are considered (1 × 1 × 1, 2 × 2 × 2, and 3 × 3 × 3).

The experimentally measured dielectric constant of pristine HKUST-1 is 1.717 ± 0.003, in keeping with the p-DFT prediction.^9^ This value was used as a benchmark to optimize the Thole damping parameter *a* (see equations S3 and S4 in Supporting Information). For benzene, *a* = 2.6 was estimated and is typically used for simulations of other small-sized organic molecules,^41^ amino acids,^61^ and ions in water.^37^ Using this value, a static dielectric constant of 1.88 is calculated for pristine HKUST-1, which is 9.9% larger than the experimental and DFT-calculated data.^9^ It is important to note that the Thole terms have no direct quantum mechanical (QM) analogue, and to the best of our knowledge, there are no relevant studies to date, reporting the application of the Drude model for MOFs. Thus, we repeated MD simulations, scanning *a* in the range from 2.0 to 3.0 (Figure 2).

**Figure 2:**
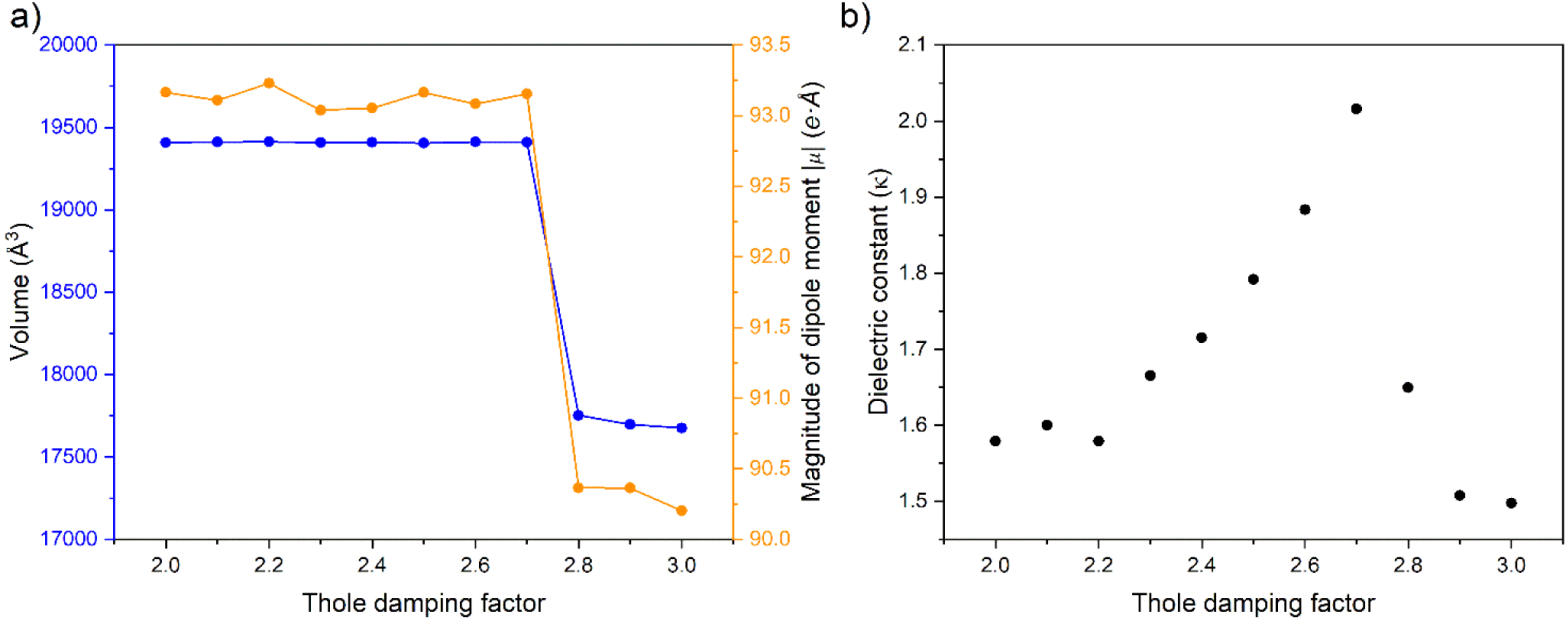
(a) The dependence of volume and collective dipole moment of the pristine HKUST-1 unit cell on the Thole damping factor; b) Dielectric constant obtained by scanning the damping factor from 2.0 to 3.0.

For MOF-type materials, the harmonic bonds stretch around their equilibrium distance, *r*_*0*_, which is *r*_*ij*_ in equation S1 for all atom pairs. Given that, the value of *r*_*ij*_ is relatively fixed for each 1–2 and 1–3 interaction (Figure S2), it is instructive to view the screening coefficient (*S*_*ij*_) as a function of the damping factor with equation S4 in Figure S3. In general, *S*_*ij*_ increases with stronger *a* resulting in smaller Coulombic screening, although the effect varies for each atom pair. The C1–C2 interaction is highly sensitive to variations in *a*, while C–Cu is markedly less affected over the damping factor range from 2.0 to 3.0.

As shown in Figure 2a, the volume calculated with DO-UFF4MOF and a damping factor from 2.0 to 2.7 is close to the expected volume, and the same applies to the corresponding dipole moment, but both abruptly drop for damping factors larger than 2.8. This is caused by an unphysical bond length obtained for each C–C pair and an unreasonably long DC-DP pair distance for carbon when using the set of parameters given by Lamoureux and Roux (Figure 2b, Tables S4 and S5). Likely, the unphysical C–C interaction is a consequence of the DC-DP distance of C atoms because it was, instead, well reproduced in the NP model. Given the direct correlation between the force constant *K*_*D*_ and the induced dipole response, parameter *a* must be scaled concomitantly with *K*_*D*_. This interdependence means that the dielectric response profiles obtained for different values of *a* are not universal; rather, they are intrinsically linked to the specific set of values of *K*_*D*_ for which *a* was derived, leading to a family of distinct response curves. Therefore, the suitable range for the *a* value is up to 2.7 for HKUST-1.^37^ More precisely, *a* = 2.4 returns the static dielectric constant (1.715) closest to the experimental value and to the DFT prediction, with well-controlled motion of DC-DPs and preservation of the cooperative response of the ordered framework. Therefore, *a* = 2.4 was selected for the following simulations.^9^ Noteworthy, we cannot generalize this parameter for all MOFs or, in any case, MOFs based on the BTC linker. Nevertheless, the optimization procedure reported here is quite straightforward, and this strategy could be adopted for the calculation of other MOFs.

### 3.2 Defect Simulation

To test the suitability of the force field for defective HKUST-1, we first calculated, quantum mechanically (at the DFT level), the electronic polarizabilities of [Cu_2_(H_2_BTC)_4_], [Cu_2_(H_2_BTC)_3_(FA)], [Cu_2_ *o*-(H_2_BTC)_2_(FA)_2_], [Cu_2_ *p*-(H_2_BTC)_2_(FA)_2_], and [Cu_2_(H_2_BTC)­(FA)_3_] fragments (Figure S4) and their partition in terms of atomic contributions. These fragments address the local perturbation of FA modulators. The atomic polarizabilities of carbon and oxygen atoms in the BTC carboxylate group are very similar in pristine and defective fragments (Table S6), whereas copper atoms in defective fragments feature lower polarizability compared with pristine HKUST. This is a consequence of the lower overall polarizability of a smaller species like FA compared to BTC, which therefore reduces the polarizability of the metals to which they are bonded. In formate linkers, oxygen atoms are slightly less polarizable than their counterparts in BTC. This implies that the defect produced by the modulator reduces the polarizability of the systems in three ways: a) six C atoms of the aromatic BTC ring are missing; b) the carboxylate group of FA is less polarizable; c) the lower polarizability of FA induces lower polarizability in the rest of the fragment (especially at the Cu atoms). The most important effect is a), because removing six C atoms of BTC corresponds to reducing by ca. 66 Bohr^3^ the polarizability. In a unit cell, there are 32 BTC linkers. In the case of 50% defect concentration, the missing linker would produce a κ reduction of ca. 0.11 units. The other two effects (smaller polarizability of the FA carboxylate group compared with BTC and reduced polarizability of Cu ions) are instead much less relevant. For this reason, in MD simulations of defective HKUST-1, we maintained the same set of parameters of the pristine model for all atoms atoms of BTC or FA. Therefore, only factor a) is inherently accounted for in the simulation.

We then calculated the dielectric constant of pristine HKUST-1 using the DO-UFF4MOF model, comparing the simulated 1 × 1 × 1 cell against the 2 × 2 × 2 and 3 × 3 × 3 supercells. The dielectric constant did not change appreciably (Table S7); therefore, underlying size effects can be safely neglected. For this reason, we chose a 2 × 2 × 2 simulation box size for all subsequent calculations on defective structures. We tested the trend of the dielectric constant with increasing defect concentration by calculating models with 5%, 10%, 20%, 30%, 40%, and 50% defect concentrations, respectively. A 50% defective structure has not been accessed experimentally so far, and of course, its hypothetical stability strongly depends on the distribution of the defects, because paddlewheel units with all four BTC replaced by FA would imply a structural collapse. Thus, the 50% defect concentration is only an idealized case, used here for better extrapolation of the trend. Moreover, all of the defective models are generated artificially to avoid disconnection between the nodes. The rigidity /stability is expected to be reduced with more defects in the frameworks, resulting in low dielectric improvements.^34^

The calculations clearly indicate a sharp decrease in the dielectric constant as the concentration of defects increases, starting from a reduction of 0.03 for low defect density (5%) up to 0.20 for 50% missing linker defects, compared with the pristine HKUST-1 (Table S7). Eventually, the dielectric constant predicted with 50% defects was as low as 1.51. The 0.2 reduction is approximately two times larger than what would be predicted based solely on the atoms formally removed (see discussion above), and it indicates a cooperative effect in the extended structure.

We then considered the distribution of defects and calculated, for each defect concentration, three configurations with random defect distribution. An almost invariant dielectric constant was calculated (within a 1% spread), as shown in Figure 3a. A full statistical analysis of the configurational space is computationally prohibitive; anyway, this limited exploration seems to indicate that the spatial arrangement of the defect sites has a minor effect on the dielectric constant, as also observed for predictions of thermal conductivity and elastic constant in defective HKUST-1.^32,34^ Nevertheless, different defect distributions may lead to more significant variations and would need further investigation.

**Figure 3:**
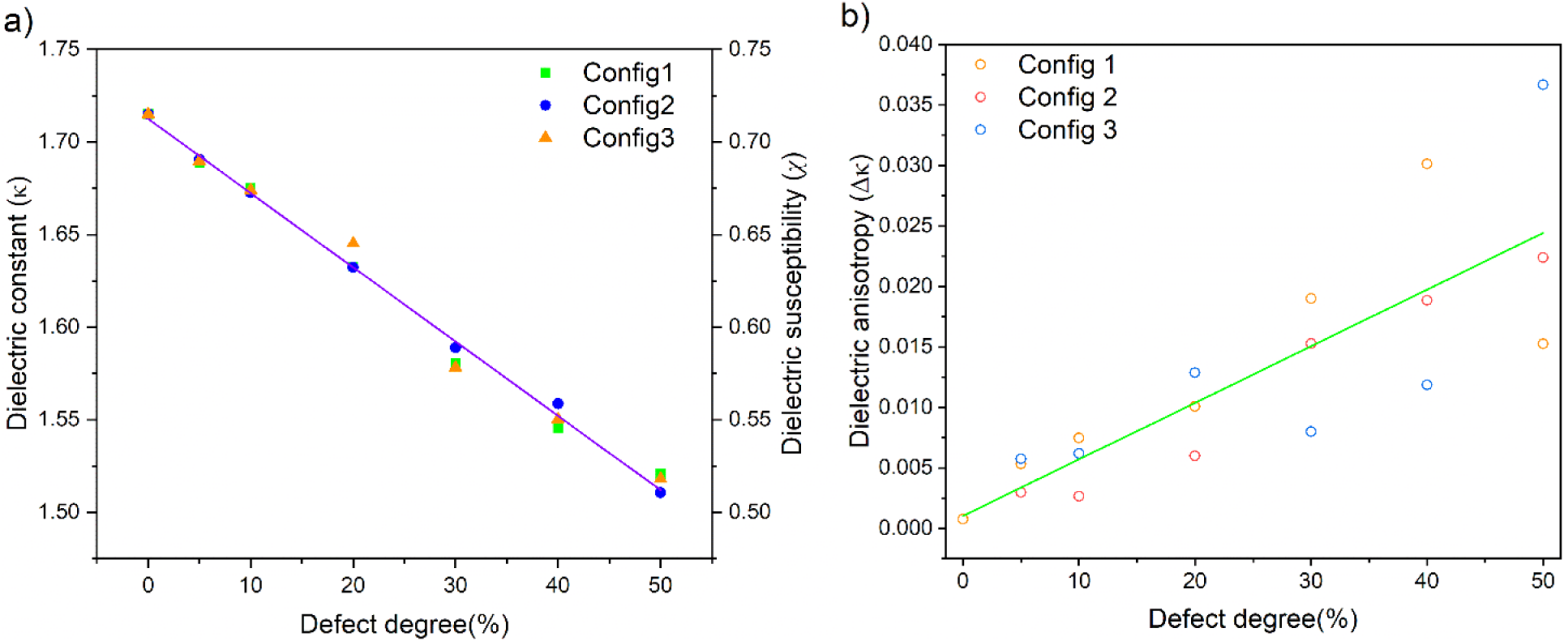
(a) Dielectric constant and susceptibility of HKUST-1 calculated with a 2 × 2 × 2 supercell as a function of defect concentration for three configurations (the purple line shows the linear regression fitting); (b) The anisotropy of the dielectric constant along *x*, *y,* and *z* directions for defective HKUST-1 as a function of defect concentrations (the green line shows the linear regression fitting).

The introduction of defects implies more possibilities to deviate from the ideal cubic symmetry, despite the overall framework retaining the original network connectivity. Therefore, the dielectric constant, which is perfectly isotropic in the ideal defect-free HKUST-1, becomes slightly anisotropic. This was estimated by $\Delta \kappa ={\{\frac{1}{2}[3\mathrm{Tr}(\kappa^{2})-\left(\mathrm{Tr}\kappa {)}^{2}\right]\}}^{1/2}$ ,^62^ showing a monotonic increase with the defect concentration, reaching the maximum Δκ*_r_* at the 50% defect model (see Figure 3b). The anisotropy spread between different configurations is narrow for low defect concentrations, but becomes wider with more defects. This may be explained by considering that a low concentration of defects implies a smaller probability of localizing them along one direction, whereas with higher defect concentrations, one may also find an inhomogeneous distribution.

As shown in Figure 3a, the correlation between the dielectric constant and defect degree is linear. Considering eq 2, κ decreases with lower polarizability (lower spread of the dipole moments) and/or larger material volume *V*. From calculations with the DO-UFF4MOF force field (FF_NPT_DO model), the volume contraction is minimal (only 2% with 50% defect) and just slightly larger with the NP-UFF4MOF (FF_EM_NP model) (see Table S3). Because the replacement of BTC with FA implies removing six C atoms (corresponding to ca. 10% of the pristine volume for a 50% defective structure), the small contraction of the framework volume implies a larger portion of empty volume. The simulations do not predict a collapse of the structure even with a 50% defect. Of course, the MD simulations carried out without a reactive force field cannot predict possible phase separation, and therefore it cannot be guaranteed that a preparation including 50% of formic acid instead of H_3_BTC would, in fact, lead to a 50% defective structure. Nonetheless, the simulation with high defect concentration is useful for better highlighting the correlation between defective structure and dielectric properties. Here, it is interesting to note that the defect does not significantly reduce the overall volume and therefore does not act against a lower dielectric constant.

From eq 2, the other main variable affecting κ is the polarizability (*α* = Δ*μ*/*E*). The simulations predict a reduction, compared to pristine HKUST-1, of 3.7% and 29.3% for 5% and 50% defect concentration, respectively (see Figure 4) with a rather linear decrease. As anticipated above, the missing C atoms contribute only 11% of the polarizability. This means that the introduction of linker defects reduces the polarizability more than what is expected from missing atoms, while substantially preserving the framework volume. These two combined effects are extremely favorable and indicate that the dielectric constant shrinks enormously with increasing defect concentration. This is also evident if we compare the polarizability and the mass density. The latter is reduced by only 7.5% at 50% defect concentration, whereas the polarizability is reduced by nearly 30%.

**Figure 4:**
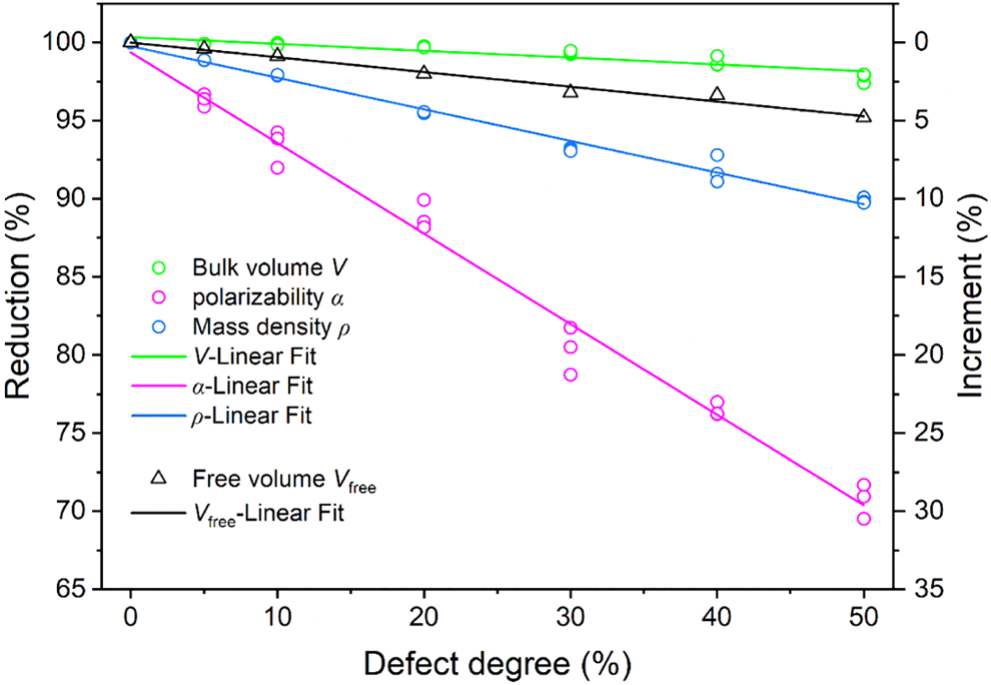
The reductions in polarizability, mass density, and bulk volume (circles, scale on the left y-axis), and the increment in free volume (triangles, reverse scale on the right y-axis) are calculated for the 2 × 2 × 2 cell as a function of the defect amount. For each defect concentration, three configurations are plotted together with linear regression lines.

Therefore, it is demonstrated that by removing atoms, one does not remove just the associated electrons from the polarizability but also their influence on the electron polarizability of the remaining atoms. This proves that the defect strategy is a very effective way to modulate the polarization response by breaking the long-range cooperative mode.

### 3.3 Frequency Dependence of the Bulk Polarization

The frequency dependence of the dielectric constant is one of the fundamental characteristics of polarizable materials, arising from the dynamic behavior of various microscopic polarization mechanisms. Unfortunately, the Drude oscillators are still massive and not perfectly adequate for the calculation of frequency-dependent properties,^37^ which could be a possible strategy for further model refinement. Therefore, the charged NP models were adopted for calculations of the dielectric spectrum in the THz range. It should be acknowledged that the NP model does not explicitly include ionic/electronic polarizations.^63^ As a result, the calculated dielectric response in the THz range may be underestimated. The complex dielectric function is *κ̃*(*ω*) = *κ′*(*ω*)+ *iκ″*(*ω*), comprising the real *κ′′* and the imaginary component *κ″*. The latter represents the frequency-dependent dissipation of energy. The dielectric spectra were calculated over a wide frequency range from the Fourier transformation of the current autocorrelation function ⟨*J*(0) · ***J*** (*t*)⟩ (CACF) with the equations (Figure S5):

$$\kappa \left(\omega \right)=\varepsilon_{\infty }+\frac{4\pi }{3Vk_{B}Ti\omega }\overset{\infty }{\underset{0}{\int }}e^{i\omega t}\left\langle J\left(0\right)\cdot J\left(t\right)\right\rangle dt$$ 3

where *ε*_∞_ is the electronic relative dielectric permittivity at infinite frequency, *V* is volume, *k*_*B*_ is the Boltzmann constant, *T* is temperature, and the angular frequency is *ω* = 2*πν*. CACF is equivalent to the dipole moment autocorrelation function in a neutral system, but it converges more rapidly. The current ***J*** is calculated from $\underset{a}{\sum }q^{a}v_{i}^{a}$ , in which *q_a_* is the atomic charge and $v_{i}^{a}$ is the velocity vector along the same Cartesian component. The trajectories were recorded after 50 ps of NVT equilibration with 2 ns integration; the collective current was sampled every 5 fs, and only the last 1 ns of the CACF was used to obtain the dielectric spectrum to avoid the influence of the signal-to-noise ratio (Figure S6). The peaks in the spectra of pristine HKUST-1 until 30 THz are in good agreement with experimental studies,^13^ while the peaks predicted in the range of 55 THz to 65 THz in Figure 5 should occur at around 40 THz. In fact, the combination of charge distribution and FF parameters could not properly capture the electronic structures. However, these unmatched peaks are attributed to C–C and C–O vibrational modes, which contribute marginally to the entire polarization, and the absolute position of the peaks is not critical for the present study that investigates the influence of defects on the frequency dependence more than on the absolute frequency.

**Figure 5:**
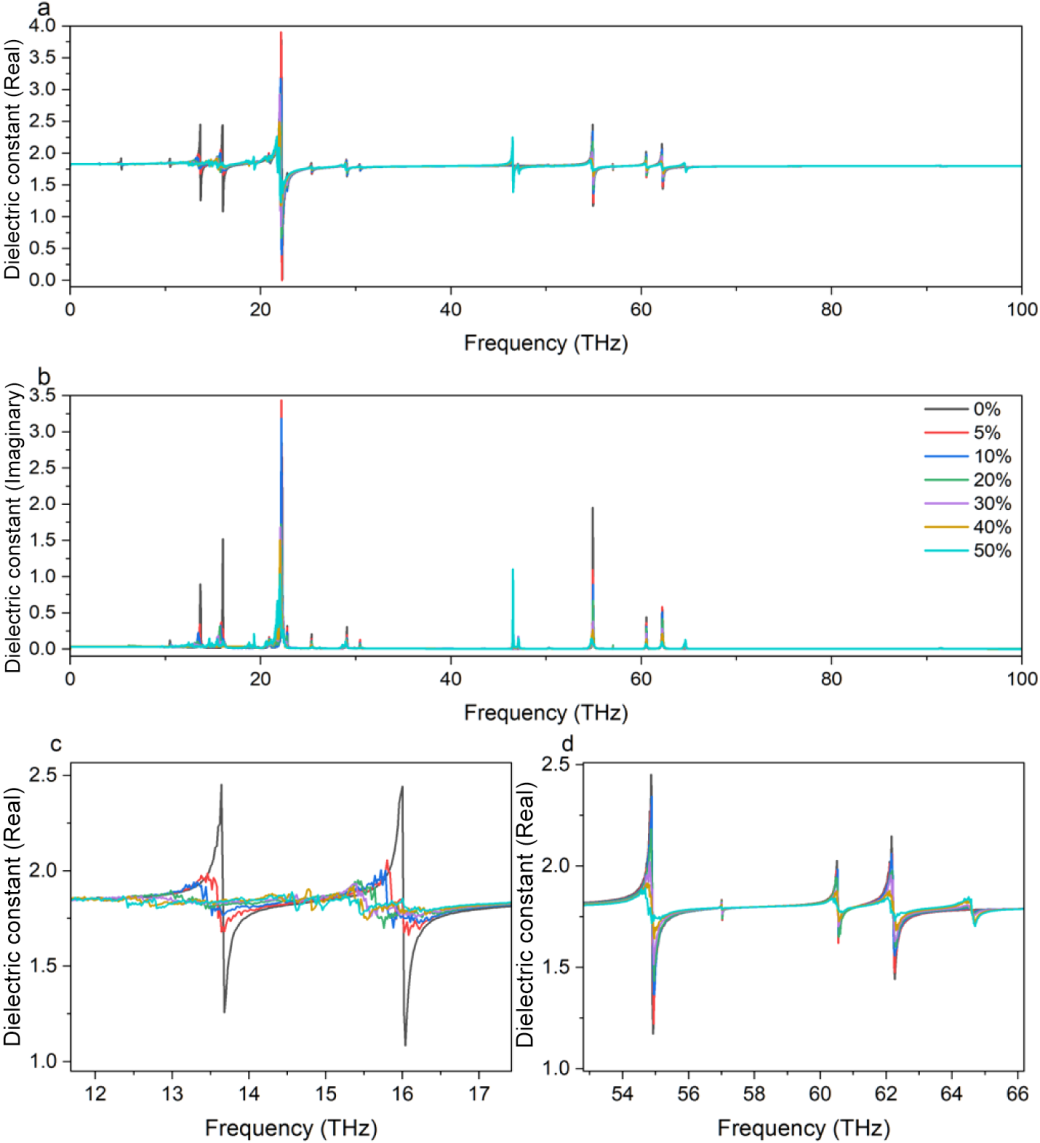
Real (a) and imaginary (b) part spectra of pristine and defect models up to 100 THz. Panels (c) and (d) show enlargements of the real spectrum over 12–17 THz and 54–66 THz, respectively.

The same method was applied to all defect models. The characteristic peaks in defect spectra cannot distinguish the random distribution because the dipole moments were recorded as a whole (Figure S7), and the dielectric relaxation appears to be less affected by the distribution of defect sites with the limited number of models. Further exploration is needed with more models. In Figure 6, we observe a reduction in peak intensity upon defect introduction, which might primarily stem from a decrease in the polarizing units. Meanwhile, the characteristic peaks shift to lower frequencies (Figure 5c and 5d), suggesting that the defects could alter the polarization dynamics. A visual inspection of the vibrational modes over the frequency range for the pristine structure indicates that the libration of the benzene rings decreases at higher frequencies compared to the low-frequency region, while the copper centers show only a modest change. It is therefore expected that, in the defective model—where defect sites arise from the replacement of benzene rings—the vibrational spectrum shifts toward lower frequencies. Moreover, this shift becomes more pronounced with increasing defect concentration, leading to further redistribution of modes into the low-frequency region. Moreover, the introduction of structural defects would seem to lead to more rapid dipole moment fluctuations because they are less constrained than in the pristine (highly ordered) structure.

**Figure 6:**
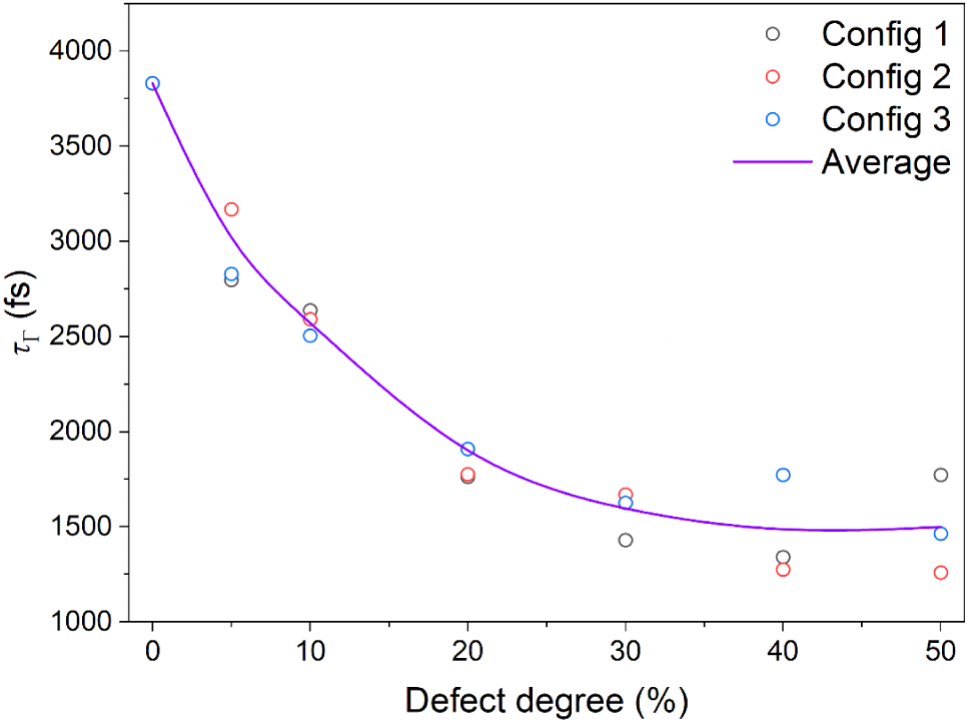
Median relaxation times of the pristine and defective crystals with three configurations are also shown. The purple line represents the average value.

Overall, the defects speculatively break down strong and collective modes into a broader distribution of weaker, localized modes. To prove this, we analyzed the timescale of the normalized current autocorrelation function $\phi \left(t\right)=\frac{\langle J\left(0\right)\cdot J\left(t\right)\rangle }{\langle J\left(0\right)\cdot J\left(0\right)\rangle }$ in Figure S6, in which the signals with sharp and irregular side peaks were treated with a Hilbert transform for envelope calculation (Figure S8), followed by a moving average filter for the sake of attenuating high-frequency noise. Then, the data were fitted with the Kohlrausch/Williams/Watts function (KWW function) for non-Debye relaxation:^64^

4 $$\phi \left(t\right)=\exp\left[-{(\frac{t}{\tau_{\mathrm{KWW}}})}^{\beta_{\mathrm{KWW}}}\right]$$

where *τ*_KWW_ is the relaxation time, and the stretching parameter *β*_KWW_ with 0 < *β*_KWW_ < 1, leads to an asymmetric broadening of *ϕ*(*t*) compared with the Debye-type decay.

The dielectric relaxation depends on fluctuations of dipoles due to molecules or fragments oscillating in keeping with the electric field. Meanwhile, the drift motion of mobile charge carriers (electrons, ions, or charged defects) causes conductive contributions to the dielectric response.^64^ For HKUST-1 derivatives, *β*_KWW_ parameter varies from 0.35 to 0.50, yielding a good fitting (Table S8). Since the distribution of relaxation times is the probability density function of the relaxation modes, and it is not comparable when the stretched exponents are not equal, the comparison is made through the median relaxation time (*τ*_Γ_), as suggested by Shamblin et al.:^65^

5 $$\tau_{\Gamma }=\frac{\tau_{\mathrm{KWW}}}{\beta_{\mathrm{KWW}}}\Gamma \left(\frac{1}{\beta_{\mathrm{KWW}}}\right)$$

where Γ is the gamma distribution function. The relaxation time for pristine HKUST-1 was predicted as 1.5 ps with *β*_KWW_ of 0.45, which is in a reliable range compared with the reported hybrid perovskites and disordered liquid systems.^66,67^ The relaxation times *τ*_Γ_ as a function of defect degree indicate that even a small concentration of defects has a significant contribution. A 5% defect concentration reduces *τ*_Γ_ by 23.5% (Figure 6), and there is sustained accelerating growth with more defects introduced until 30%. Then, the relaxation time remains constant for all highly defective HKUST-1 structures up to 50%, which is 61.9% faster than the pristine HKUST-1. Furthermore, the distribution of *τ*_Γ_ for different configurations becomes wider in the highly defective cases, which is consistent with the above anisotropy analysis. The defects could locally perturb the electrostatic environment by breaking symmetry and weakening cooperative polarization constraints, enabling faster dipole reorientation and hence accelerating dielectric relaxation at low defect concentrations. As defect density increases, the growing overlap of defect-induced local fields seems to lead to saturation of the relaxation enhancement. Beyond this point, strong structural and electrostatic disorder introduces competing relaxation pathways to some extent, which suppress collective dipolar motion and counterbalance the initial acceleration, causing the relaxation rate to level off.

## 4 Conclusions

In this study, we investigated the effect of missing linker defect concentration on the dielectric constant of HKUST-1 using classical molecular dynamics simulations. We adapted the thermalized Drude oscillators to the generic UFF4MOF force field, incorporating extended charge equilibration methods to capture the response of molecular dipole moments to an external electric field. The Thole damping factor (essential to avoid the so-called polarization catastrophe) was optimized to properly reproduce the dielectric constant of pristine HKUST-1.

The model was then applied to simulate the behavior of HKUST-1 derivatives with missing linker defects at concentrations of up to 50%, introduced via random spatial arrangements and replacement of BTC linkers with FA ligands. This preserves the electroneutrality without imposing missing cluster nodes, while at the same time it reduces the linkages of the framework (FA being able to bind only one Cu paddlewheel instead of three like BTC). The dielectric constant was found to decrease linearly with an increasing number of defects, reaching a minimum of 1.51 at 50% defect concentration (the largest defect concentration calculated here calculated), which is a significant reduction compared to the pristine material. This interesting and very promising behavior arises primarily from a strong decrease in the system’s polarizability, whereas the volume is almost unaffected by the increasing number of defects. The approximately linear correlation between the dielectric constant and defect concentration enables straightforward predictability. These findings also offer a plausible explanation for discrepancies between experimentally measured and computationally predicted dielectric constants of MOFs, especially when theoretical models assume defect-free crystals. Very often, instead, these discrepancies are attributed solely to air gaps in the microstructures.

In addition, we computed the dielectric spectrum over a wide frequency range. Our simulations predict a significant decrease in peak intensity and notable frequency shifts as a function of defect concentration. This suggests that defect concentrations in a sample could be estimated, for example, by measuring low-frequency Raman spectra. We also evaluated dielectric relaxation across all defective HKUST-1 models and found that even low defect concentrations lead to a substantial reduction in relaxation time, with up to 61.9% acceleration at 50% defect concentration. This is likely due to the disruption of long-range polarization pathways by the defects.

This work sheds light on the structure–property relationship of dielectrics governing the dielectric response of MOFs and paves the way for designing MOFs with tailored dielectric properties through defect engineering.

## Supplementary Material



## Data Availability

Force field and coordinate files that are used for simulation and analysis are available in the data repository through https://github.com/ywang002/diel-MOFs.

## Notes

Detailed procedure of the force field parametrization and additional figures for the frequency-dependence of dielectric calculations (PDF)

## #

Department of Chemistry, Materials and Chemical Engineering, Politecnico di Milano, via Bassini 6b, 20133, Milano, Italy
