# Supplementary material for: Impact of Linker Defects on the Dielectric Properties of the Metal-Organic Framework HKUST-1: Insights from Molecular Dynamics Simulations
Source: ACS Appl Mater Interfaces. 2026 Jun 19;18(25):35557–66. doi: 10.1021/acsami.6c03219 (PMC13339010; doi:10.1021/acsami.6c03219)
Supplement: Supplementary file 1 [file am6c03219_si_001.pdf]

Supporting Information

Impact of linker defects on the dielectric properties of the Metal-organic framework HKUST-1: Insights from Molecular Dynamics Simulations

Yidian Wang[§] *and Piero Macchi*[§,*]

[§] Department of Chemistry, Materials and Chemical Engineering, Politecnico di Milano, via Bassini 6, 20133 Milano, Italy

*Corresponding author: piero.macchi@polimi.it

Force Field: The generic UFF4MOF force field is:[1]

$$U_{UFF4MOF}(r_{ij}) = \sum_{bonds} K_{ij}(r_{ij} - r_0) + \sum_{angles} K^a_{ijk}[1 - \cos(n\theta_a)]$$
$$+ \sum_{dihedrals} K_{ijkl}[1 - d\cos(2\theta_d)]$$
$$+ \sum_{impropers} K^t_{ijk}[C_0 - C_1\cos(\omega) + C_2\cos(2\omega)]$$
$$+ \sum_{i\neq j} 4\varepsilon_{ij}[(\frac{\sigma_{ij}}{r_{ij}})^{12} - (\frac{\sigma_{ij}}{r_{ij}})^{6}] + \sum_{i\neq j}\left(\frac{q_i q_j}{r_{ij}}\right) \#(S1)$$

K is the force constant for the corresponding potential terms, r_0 is equilibration structural parameters; *n* is multiplicity and θ_a is the bond angle of atoms i, j and *k*; θ_d is the dihedral angle of atoms i, j, *k* and *l*; ω is the angle between the *il* axis and the *ijk* plane; σ_{ij} and ε_{ij} are the Lennard Jones (LJ) potential parameters for the interactions between atoms *i* and *j*; *q* is a charge parameter.

In this atomistic study, DOs are meant to capture effects of electrons which instantly respond based on Born-Oppenheimer approximation to separately integrate the motion of DC and DP in MD simulations.[2] Hence, DPs are assigned a small mass and are maintained at 1 K (with a different thermostat applied to DPs from the rest of the system) to minimize their contribution to the system's kinetic energy,[2,3] and $U_{\text{self}}(r_{ij})$ term can also be the self-polarization contribution and becomes dependent on the electric field:

$$U_{\text{self},i} = \frac{1}{2}\alpha_i \vec{E}_i^2 \qquad (S2)$$

An extreme scenario may arise as atoms approaching each other to a certain limit such that head-to-tail induced dipoles diverge, causing the so called “polarization catastrophe”.[4] Such ill-behavior may occur in MD simulation during millions of integration time steps, it can be restricted by some sort of damping function that can reduce the dipole−dipole interactions from r^{-3} at short distance. Consequently, The Thole screening scheme emerged to effectively attenuate such unexcepted interaction when ultrashort distance of 1-2 and 1-3 interactions occurs. The electrostatic interactions are modulated by:

$$U_{\mathrm{elec}}(r_{ij}) = \left(\frac{q_i q_j}{r_{ij}}\right) S_{ij}(r_{ij}) \qquad (S3)$$

where r_{ij} is the distance between charged atoms i and j, and the general form of the shielding function $S_{ij}(r_{ij})$, which is written as:

$$S_{ij}(r_{ij}) = 1 - \left(1 + \frac{(a_i + a_j) r_{ij}}{2(\alpha_i \alpha_j)^{\frac{1}{6}}}\right) \mathrm{e}^{-(a_i + a_j) r_{ij}/(\alpha_i \alpha_j)^{\frac{1}{6}}} \qquad (S4)$$

where α_i and α_j are respective atomic polarizabilities, and a is Thole damping parameters that modulate the shielding strength of S_{ij}. The factor a value of 2.6, similar to the 2.089 suggested by Thole, was originally proposed to reproduce the polarizability anisotropy ratio of idealized benzene and was adopted as a universal parameter for C, N, O.[4,5] Although it is independent of individual chemical environment, it can satisfy molecular polarizabilities of some organic molecules and water.[4–8] Also, some works

aimed at fitting the polarizabilities of more other organic molecules have done to improve Drude model.[9,10]

Dielectric Constant Calculation: In general, the relative permittivity $\kappa(\omega)$ is a frequency-dependent rank-2 tensor, whose non-zero elements are determined by crystal symmetry. For orthorhombic crystals, only the diagonal elements are non-zero, while in cubic and isotropic systems, all three diagonal elements are equal. Therefore, one could look at the isotropic dielectric constant, instead of the anisotropic contributions:

$$\kappa = \frac{\kappa_{xx}+\kappa_{yy}+\kappa_{zz}}{3} \qquad (S5)$$

Median Relaxation Time (frequency-dependency): Since the distribution of relaxation times as the probability density function of the relaxation modes, the equation 5 can also be written as:

$$\phi = \int_{-\infty}^{+\infty} f(\tau)\exp\left(-\frac{t}{\tau}\right)\partial\tau \qquad (S6)$$

where $f(\tau)$ is the function that describes the distribution of relaxation times with assumption of the mean relaxation time remains constant throughout the relaxation process.

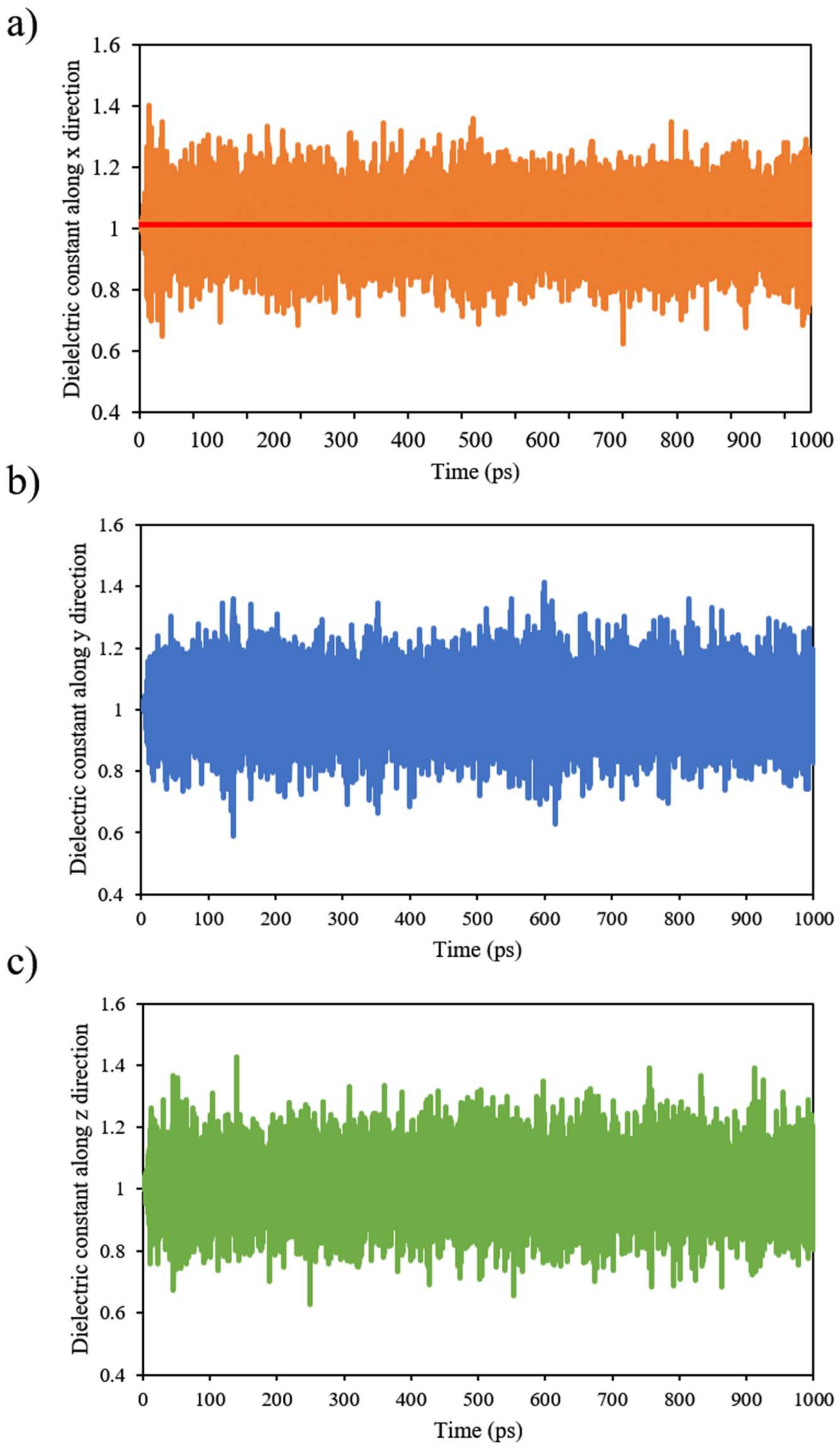

Figure S1. The dielectric constant along a) *x*, b) *y* and c) *z* directions under electric field of 0.025711 Volt/Å. 1 ns simulation is sampled every 0.25 fs, and red line in a) is the average value of dielectric constant. The average dielectric value for *x*, *y*, *z* is 1.01, 1.01, 1.01, respectively.

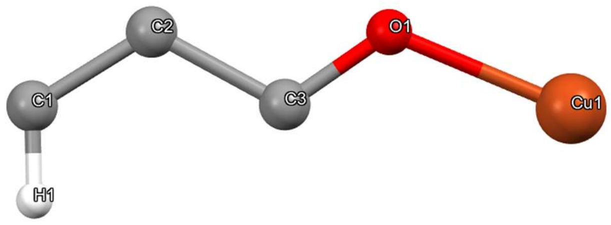

Figure S2. The asymmetric unit of HKUST-1.

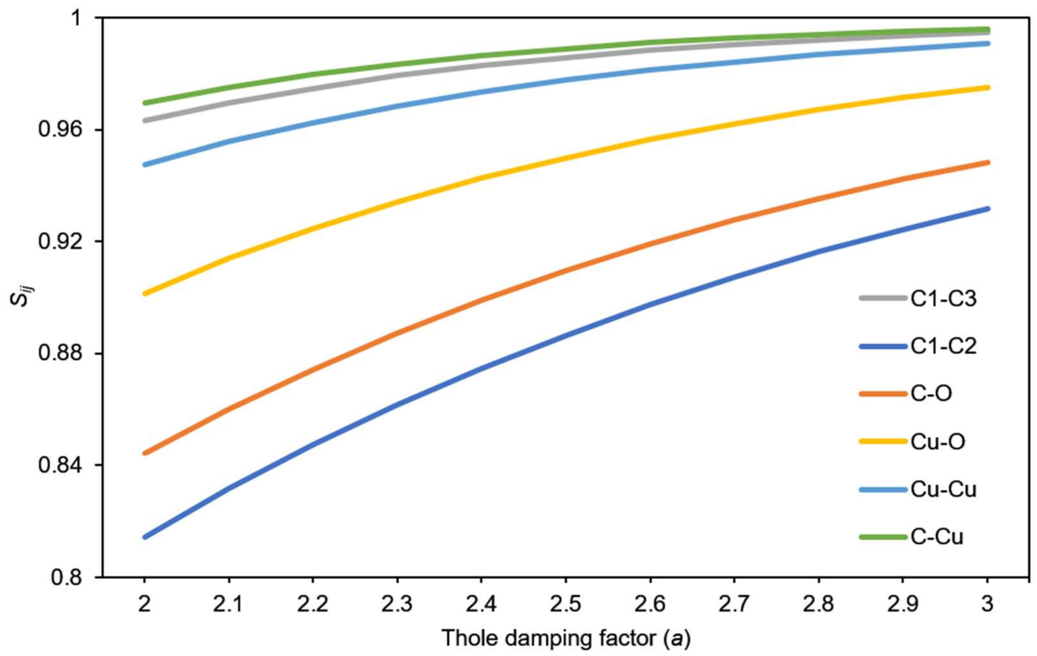

Figure S3. Screening coefficient (S_{ij}) as function of damping factor (a) for 1-2 and 1-3 interactions in HKUST-1 framework.

Table S1. EQeq assignment for pristine HKUST-1.

Atom type	Charge (e)
Cu	0.90
O	-0.40
C1	0.07
C2	0.35
C3	-0.12
H	0.05

Table S2. Lattice parameters of FF energy minimized and NPT relaxed pristine and defective HKUST-1, with all distances in Å.

Defect degree / Configuration	FF_EM_NP				FF_NPT_DO			
	a / Å	*b* / Å	*c* / Å	Volume ($Å^3$)	*a* / Å	*b* / Å	*c* / Å	Volume ($Å^3$)
Pristine HKUST-1								
0% (1x1x1)	26.9	26.9	26.9	19497.1	26.8	26.8	26.8	19407.2
0% (2x2x2)	53.8	53.8	53.8	155952.3	53.7	53.7	53.7	155289.0
0% (3x3x3)	81.0	81.0	81.0	532326.9	80.9	80.9	80.9	530547.1
Cell 2 x 2 x 2 with missing linker defects								
0%	53.8	53.8	53.8	155952.3	53.7	53.7	53.7	155289.0
5% / Config 1	53.8	53.8	53.8	155846.7	53.7	53.7	53.7	155189.8
5% / Config 2	53.8	53.8	53.8	155867.1	53.7	53.7	53.7	155175.8
5% / Config 3	53.8	53.8	53.8	155893.9	53.7	53.7	53.7	155141.3
10% / Config 1	53.7	53.7	53.7	155665.1	53.7	53.6	53.8	155265.9
10% / Config 2	53.7	53.8	53.8	155669.8	53.7	53.7	53.7	155101.0
10% / Config 3	53.7	53.7	53.7	155529.3	53.7	53.7	53.7	155033.4
20% / Config 1	53.7	53.7	53.6	155064.8	53.7	53.6	53.7	154843.5
20% / Config 2	53.7	53.7	53.8	155582.9	53.6	53.7	53.7	154932.0
20% / Config 3	53.7	53.7	53.7	155429.0	53.7	53.6	53.6	154800.0
30% / Config 1	53.7	53.7	53.7	155361.5	53.5	53.6	53.5	154111.7
30% / Config 2	53.7	53.7	53.7	155068.7	53.7	53.6	53.5	154227.8
30% / Config 3	53.7	53.7	53.7	155185.6	53.6	53.6	53.6	154467.9
40% / Config 1	53.5	53.3	53.3	152776.5	53.4	53.5	53.5	153072.8
40% / Config 2	53.6	53.3	53.3	152415.1	53.3	53.5	53.5	153128.0
40% / Config 3	53.7	53.3	53.2	152753.7	53.6	53.4	53.6	153957.2
50% / Config 1	52.9	52.9	52.9	148382.7	53.2	52.7	53.8	151258.2
50% / Config 2	52.8	52.8	52.8	147983.1	52.8	52.8	54.3	152011.4
50% / Config 3	52.5	52.9	53.0	147604.5	53.2	54.1	52.7	152115.0

Table S3. the average volume over three configurations after energy minimization with NP-UFF4MOF and NPT relaxation with DO-UFF4MOF.

Defect degree/Cell size	FF_EM_NP (Å^3)	FF_NPT_DO (Å^3)	Deformation (%)
Pristine HKUST-1			
0% (1 x 1 x 1)	19497.1	19407.2	-0.46
0% (2 x 2 x 2)	155952.3	155289.0	-0.42
0% (3 x 3 x 3)	532326.9	530547.1	-0.33
Cell 2 x 2 x 2 missing linker defects			
5%	155869.2	155169.0	-0.45
10%	155621.4	155133.4	-0.31
20%	155358.9	154858.5	-0.32
30%	155205.3	154269.1	-0.60
40%	152648.5	153386.0	0.48
50%	147990.1	151794.9	2.57

Table S4. Average distance of each atom pair for damping factors from 2.0 to 2.7 with DO-UFF4MOF.

Atom pair	Average distance (Å)	Average distance in NP model after EM (Å)
O_2 Cu4+2	2.02	2.03
O_2 C_R	1.28	1.28
Cu4+2 Cu4+2	2.48	2.48
C_R H_	1.09	1.08
C_R C_R	1.41	1.40
C_R C_R	1.48	1.48
O DC-DP	0.01	-
Cu DC-DP	0.01	-
C DC-DP	0.02	-

Table S5. Average distance of each atom pair for damping factors from 2.8 to 3.0 with DO-UFF4MOF.

Atom pair	Average distance (Å)	Average distance in NP model after EM (Å)
O_2 Cu4+2	2.02	2.03
O_2 C_R	1.29	1.28
Cu4+2 Cu4+2	2.48	2.48
C_R H_	1.09	1.08
C_R C_R	1.20	1.40
C_R C_R	1.52	1.48
O DC-DP	0.01	-
Cu DC-DP	0.01	-
C DC-DP	0.65	-

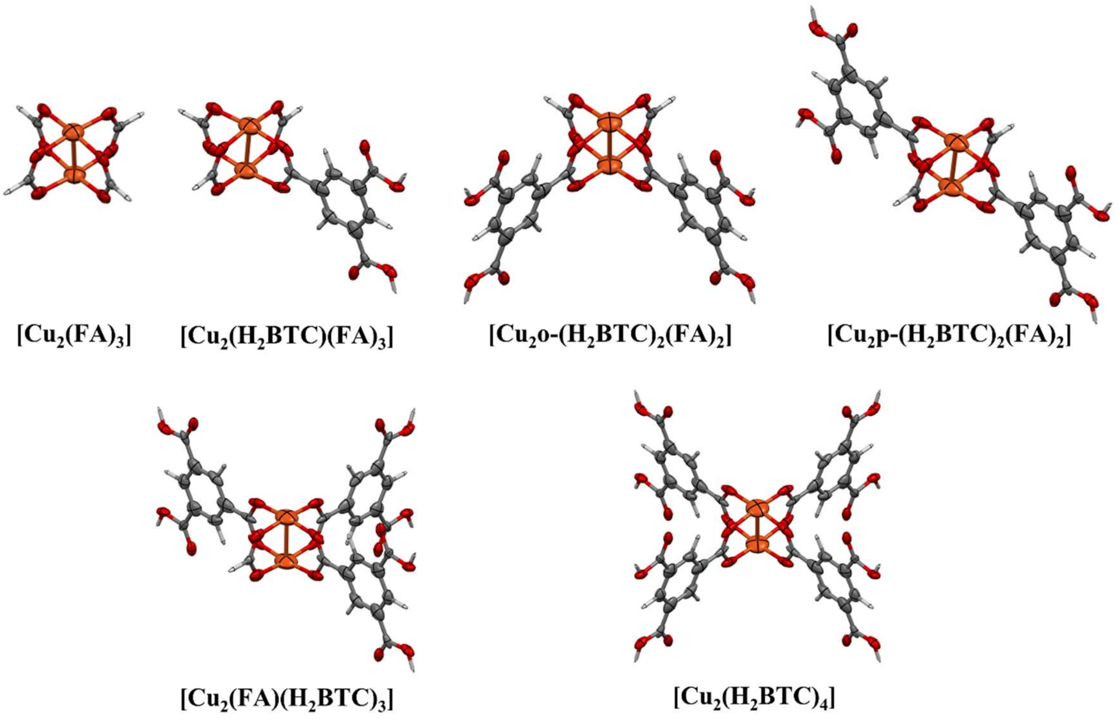

Figure S4. Calculated atomic polarizabilities (represented as ellipsoids) for closed-shell fragments of $[Cu_2(H_2BTC)_4]$, $[Cu_2(H_2BTC)_3(FA)]$, $[Cu_2$*o*-$(H_2BTC)_2(FA)_2]$, $[Cu_2$*p*-$(H_2BTC)_2(FA)_2]$, $[Cu_2(H_2BTC)(FA)_3]$, and $[Cu_2(FA)_4]$.

Color code: orange for Cu, grey for C, red for O and white for H.

Table S6. Calculated isotropic atomic polarizabilities ($bohr^3$) for selected atoms of the fragments shown in Figure S4. O refers to carboxylate oxygen atoms coordinated to Cu^{2+} ions (averaged among BTC or FA linkers); C refers to C atoms of the carboxylate groups coordinated to Cu^{2+}; C_{bz} refers to C atoms of the aromatic ring in BTC.

	$Cu_2(H_2BTC)_4$	$Cu_2(H_2BTC)_3(FA)$	Cu_2*o*-$(H_2BTC)_2(FA)_2$	Cu_2*p*-$(H_2BTC)_2(FA)_2$	$Cu_2(H_2BTC)(FA)_3$	$Cu_2(FA)_4$
Cu^{2+}	21.4	19.7	18.2	18.4	16.9	15.7
O (BTC)	11.0	10.9	10.2	11.0	10.2	-
O (FA)	-	9.5	9.6	8.9	9.2	9.1
C1 (BTC)	8.7	8.6	8.3	8.8	8.3	-
C1 (FA)	-	5.1	5.2	5.0	5.1	5.1
C_{bz} (BTC)	11.0	11.0	11.1	11.2	10.9	-

Table S7. The dielectric constant and susceptibility of DO-UFF4MOF models in various defect degrees with missing linker defects.

Defect degree	Dielectric constant(κ)	Dielectric susceptibility(χ)	χ reduced by (%)
0% (1 X 1 X 1)	1.72	0.72	-
0% (2 X 2 X 2)	1.71	0.71	-
0% (3 X 3 X 3)	1.71	0.71	-
5%	1.69	0.69	-2.8
10%	1.66	0.66	-7.0
20%	1.63	0.63	-11.3
30%	1.56	0.56	-21.1
40%	1.54	0.54	-23.9
50%	1.50	0.50	-29.6

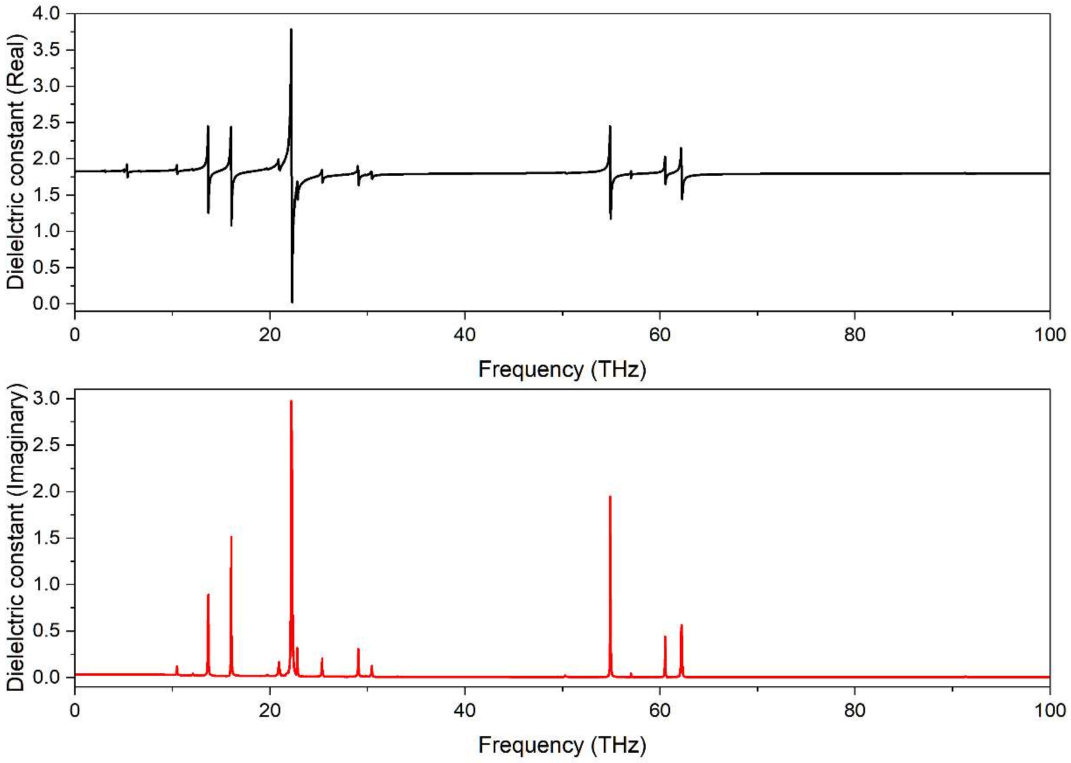

Figure S5. Dielectric spectra of pristine HKUST-1, real (black line) and imaginary (red line) parts are shown.

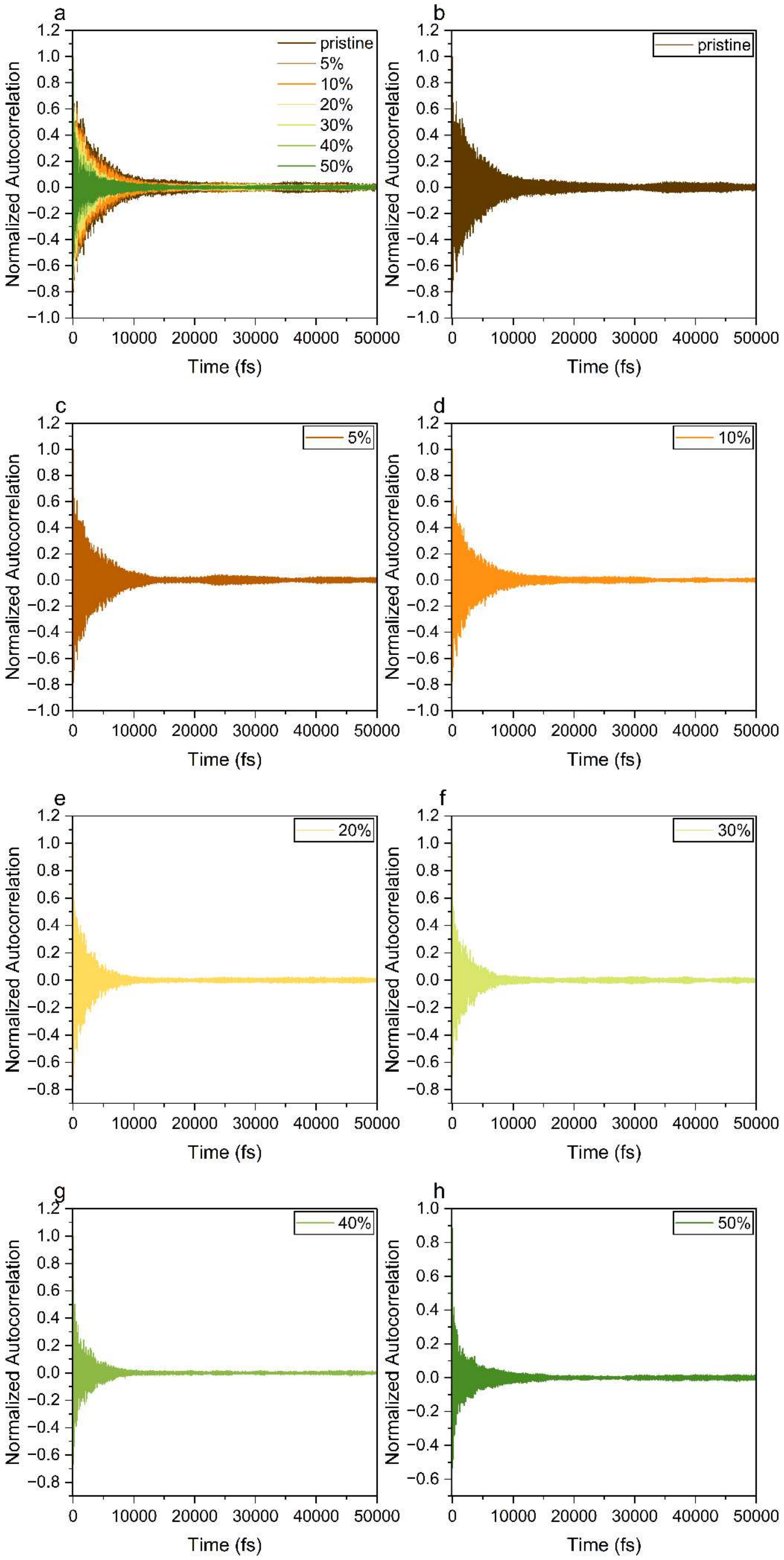
a
b
c
d
e
f
g
h
Normalized Autocorrelation
Time (fs)
pristine
5%
10%
20%
30%
40%
50%

Figure S6. Normalized current autocorrelation (CACF) as a function of correlation time (a) for pristine (b), 5% (c), 10% (d), 20% (e) 30% (f) 40% (g) and 50% (h).

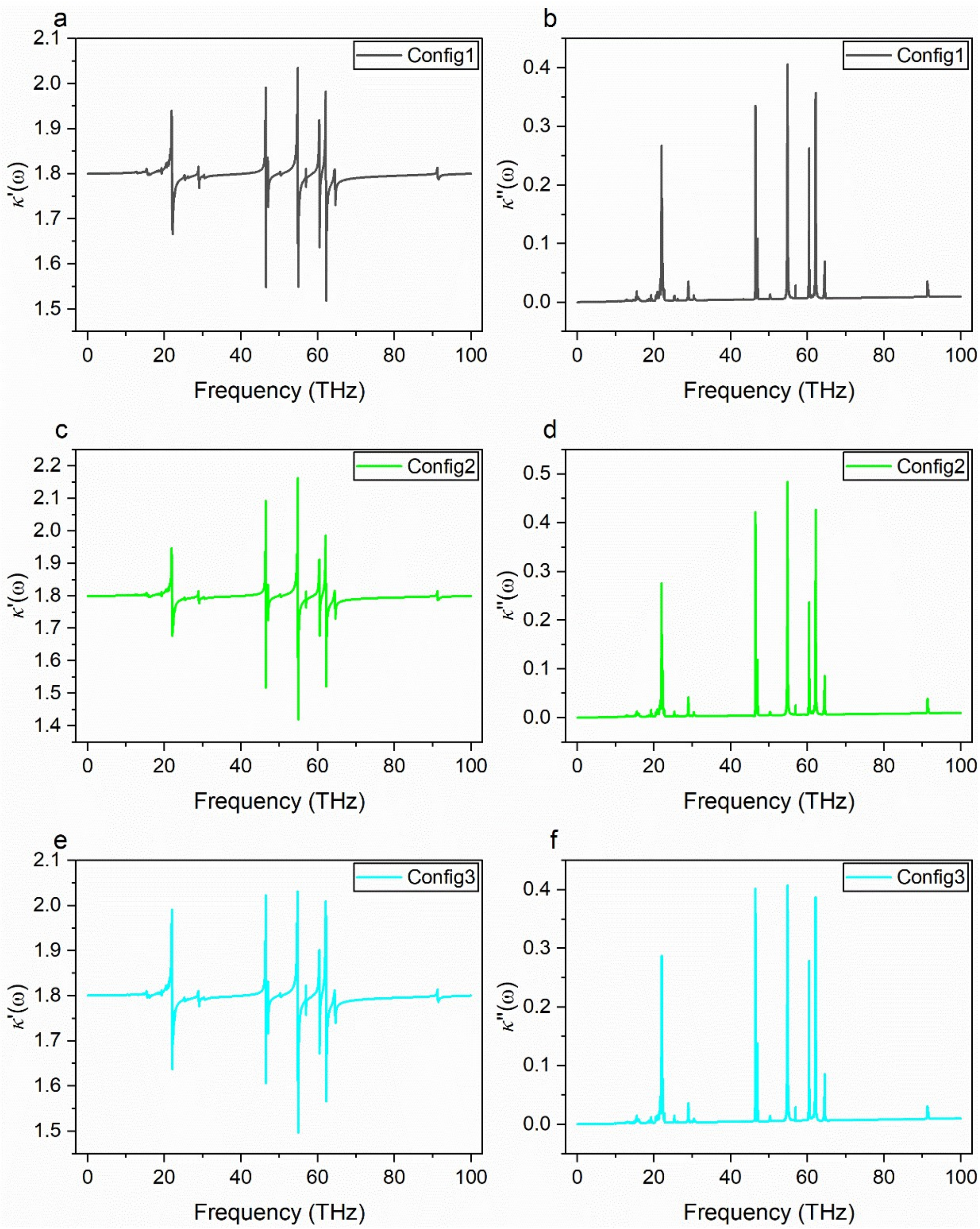

Figure S7. Frequency dependent dielectric spectra for 30% models with different defect distributions, real part for configuration 1 (a), configuration 2 (c), and

configuration 3 (e); and imaginary part for configuration 1 (b), configuration 2 (d), and configuration 3 (f).

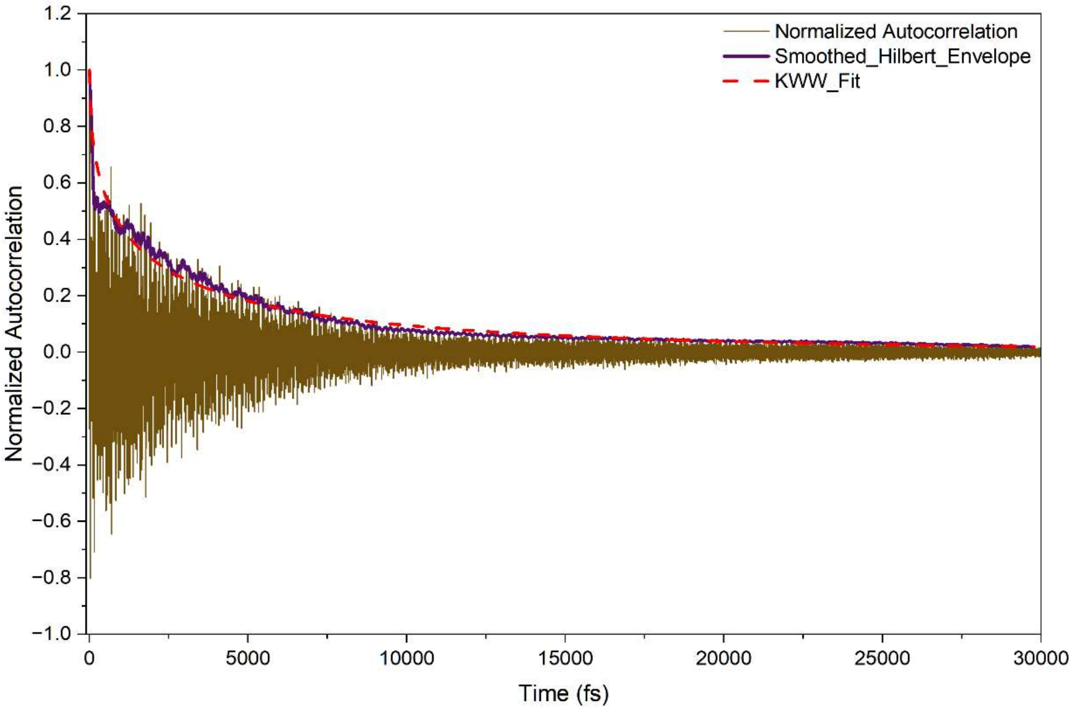

Figure S8. Normalized current autocorrelation as a function of correlation time for pristine (brown line), smoothed envelop from Hilbert transformation (purple line) and KWW fitting curve (red dash line).

Table S8. Fitted parameters of $\Phi(t) = \exp\left[-\left(\frac{t}{\tau_{\mathrm{KWW}}}\right)^{\beta_{\mathrm{KWW}}}\right]$ for pristine and defective models with three configurations in random defect sites, and their median relaxation time by $\tau_{\Gamma} = \frac{\tau_{\mathrm{KWW}}}{\beta_{\mathrm{KWW}}}\Gamma\left(\frac{1}{\beta_{\mathrm{KWW}}}\right)$.

Defect degree	τ_{KWW} (fs)	β_{KWW}	τ_{Γ} (fs)
Pristine	1545.80 ± 0.60	0.45 ± 0.01	3831.41 ± 0.82
5%Config 1	1293.48 ± 0.53	0.48 ± 0.01	2797.53 ± 0.43
5%Config 2	1403.91± 0.53	0.47 ± 0.01	3168.87 ± 0.16
5%Config 3	1308.11 ± 0.52	0.48 ± 0.01	2829.17 ± 0.33
10%Config 1	1116.92 ± 0.36	0.46 ± 0.01	2638.07 ± 0.64
10%Config 2	1096.89 ± 0.41	0.46 ± 0.01	2590.76 ± 0.96
10%Config 3	1109.55 ± 0.40	0.47 ± 0.01	2504.45 ± 0.87
20%Config 1	814.76 ± 0.34	0.48 ± 0.01	1762.16 ± 0.74
20%Config 2	716.82 ± 0.32	0.45 ± 0.01	1776.70 ± 0.21
20%Config 3	808.24 ± 0.32	0.46 ± 0.01	1908.99 ± 0.22
30%Config 1	577.33 ± 0.22	0.45 ± 0.01	1430.96 ± 0.21
30%Config 2	571.79 ± 0.22	0.42± 0.01	1670.35 ± 0.23
30%Config 3	590.27 ± 0.27	0.43 ± 0.01	1626.63 ± 0.45
40%Config 1	403.57 ± 0.20	0.40 ± 0.01	1341.20 ± 0.37
40%Config 2	463.13 ± 0.17	0.43 ± 0.01	1276.26 ± 0.94
40%Config 3	460.27 ± 0.19	0.38 ± 0.01	1773.58 ± 0.21
50%Config 1	352.67 ± 0.24	0.35 ± 0.01	1773.62 ± 0.29
50%Config 2	405.44 ± 0.21	0.41± 0.01	1260.54 ± 0.19
50%Config 3	380.13 ± 0.20	0.38 ± 0.01	1464.77 ± 0.84

REFERENCES

(1) Addicoat, M. A.; Vankova, N.; Akter, I. F.; Heine, T. Extension of the Universal Force Field to Metal–Organic Frameworks. *J. Chem. Theory Comput.* **2014**, *10* (2), 880–891.

(2) Lamoureux, G.; Roux, B. Modeling Induced Polarization with Classical Drude Oscillators: Theory and Molecular Dynamics Simulation Algorithm. *J. Chem. Phys.* **2003**, *119* (6), 3025–3039.

(3) Schröder, C.; Steinhauser, O. Simulating Polarizable Molecular Ionic Liquids with Drude Oscillators. *J. Chem. Phys.* **2010**, *133* (15), 154511.

(4) Thole, B. T. Molecular Polarizabilities Calculated with a Modified Dipole Interaction. *Chem. Phys.* **1981**, *59* (3), 341–350.

(5) Noskov, S. Yu.; Lamoureux, G.; Roux, B. Molecular Dynamics Study of Hydration in Ethanol−Water Mixtures Using a Polarizable Force Field. *J. Phys. Chem. B* **2005**, *109* (14), 6705–6713.

(6) Liu, Z.; Timmermann, J.; Reuter, K.; Scheurer, C. Benchmarks and Dielectric Constants for Reparametrized OPLS and Polarizable Force Field Models of Chlorinated Hydrocarbons. *J. Phys. Chem. B* **2018**, *122* (2), 770–779.

(7) Lamoureux, G.; MacKerell, A. D.; Roux, B. A Simple Polarizable Model of Water Based on Classical Drude Oscillators. *J. Chem. Phys.* **2003**, *119* (10), 5185–5197.

(8) Lamoureux, G.; Harder, E.; Vorobyov, I. V.; Roux, B.; MacKerell, A. D. A Polarizable Model of Water for Molecular Dynamics Simulations of Biomolecules. *Chem. Phys. Lett.* **2006**, *418* (1–3), 245–249.

(9) Kumar, A.; Pandey, P.; Chatterjee, P.; MacKerell, A. D. Deep Neural Network Model to Predict the Electrostatic Parameters in the Polarizable Classical Drude Oscillator Force Field. *J. Chem. Theory Comput.* **2022**, *18* (3), 1711–1725.

(10) Van Duijnen, P. Th.; Swart, M. Molecular and Atomic Polarizabilities: Thole's Model Revisited. *J. Phys. Chem. A* **1998**, *102* (14), 2399–2407.
